# Efficacy of Transcutaneous 4.4 MHz Radiofrequency Diathermy versus Therapeutic Ultrasound for Pain Relief and Functional Recovery in Patients with Knee Osteoarthritis: A Randomized Controlled Study

**DOI:** 10.3390/jcm12186040

**Published:** 2023-09-18

**Authors:** Yookyung Jang, Lee Gyeong Je, Sunhee Lee, Donghyun Na, Hyekyung Shin, Jong Bum Choi, Jae Chul Koh

**Affiliations:** 1 Department of Anesthesiology and Pain Medicine, Korea University College of Medicine, Seoul 02841, Republic of Korea; gamtang@korea.ac.kr (Y.J.); jellyg0209@gmail.com (L.G.J.); sunfanooo@gmail.com (S.L.); bmssdh7942@gmail.com (D.N.); 2 Department of Anesthesiology and Pain Medicine, Ajou University School of Medicine, Suwon 16499, Republic of Korea; tlsgprdu1@naver.com

**Keywords:** knee osteoarthritis, deep heat therapy, transcutaneous radiofrequency

## Abstract

Knee osteoarthritis (KOA) is a prevalent common cause of disability and pain among adults. Transcutaneous radiofrequency (RF) diathermy and therapeutic ultrasound (US) are commonly employed treatments for addressing musculoskeletal conditions. This study aims to evaluate and compare the clinical effectiveness of transcutaneous 4.4 MHz RF diathermy and therapeutic US therapy in individuals diagnosed with KOA. A total of 108 patients with KOA were randomly assigned to either the RF or US groups. Each participant underwent a series of 10 treatment sessions over four weeks and was evaluated at different time points. The assessments included physical evaluations, vital sign measurements, the Numeric Rating Scale (NRS) for pain, Western Ontario and McMaster Universities Osteoarthritis Index (WOMAC) scores, the Lequesne index, gait analysis, the 36-Item Short Form Health Survey (SF-36), and analysis of adverse responses. Both groups showed significant differences in NRS, WOMAC scores, and Lequesne index compared to baseline values at both the 10th treatment session and the one-month follow-up assessment. However, no significant disparities were observed between the two groups at each assessment point. In the gait analysis, following the 10th treatment, the RF group showed significant changes in stride length and stride velocity compared to baseline. Four weeks after the completion of treatment, both groups exhibited significant alterations in stride length and stride velocity when compared to baseline measurements. However, regarding cadence, only the RF group exhibited a significant difference compared to baseline. The findings suggest that transcutaneous 4.4 MHz RF diathermy displays a comparable effectiveness to therapeutic US in reducing pain and enhancing functional capacity among individuals with KOA. Further research endeavors are warranted to advance the efficacy of noninvasive treatments for KOA.

## 1. Introduction

Knee osteoarthritis (KOA) stands as a prominent source of pain and disability in adults. The prevalence of this disease has increased because of increased age and obesity [1]. An estimated 240 million people worldwide are affected by KOA. Patients with KOA have a significantly poorer quality of life in comparison to healthy individuals [1]. Reduced physical activity is associated with an increase in age-adjusted mortality rate [2]. Besides surgery, treatments for KOA include education, exercise, and weight loss, complemented by non-steroidal anti-inflammatory drugs (NSAIDs), corticosteroid injections, and other adjunctive medications [1]. Different physiotherapy treatments, including thermotherapy, have been shown to assist in the treatment of KOA with fewer adverse effects than medical treatments [3]. The effectiveness of specific heat therapy modalities for managing KOA remains controversial.

Therapeutic ultrasound (US) is often used to treat musculoskeletal diseases. The application of US within the frequency spectrum ranging from 1 to 3 MHz is a common approach for providing deep heat therapy [4,5]. It has thermal and mechanical effects on target tissues, leading to increased circulation, local metabolism, and regeneration [4]. Ultrasonic energy induces molecular vibration through acoustic waves. This heightened molecular motion results in the production of frictional heat, which subsequently elevates tissue temperature. This phenomenon is known as the thermal effect. The thermal effect improves local blood flow, enzymatic activity, nerve conduction velocity, contractile activity of skeletal muscles, extensibility of collagen tissues, the pain threshold, and the reduction in muscle spasms. In addition, microscopic bubbles or cavities are formed, which are nonthermal effects. Nonthermal effects are recognized for their ability to enhance vascular wall permeability, elevate cell membrane activity, and facilitate the healing of soft tissues [6]. Therefore, therapeutic US could offer additional benefits to physical therapy regimens in alleviating symptoms for individuals with KOA, thereby making it a commonly employed approach [7].

Noninvasive transcutaneous radiofrequency (RF) diathermy entails emitting high-frequency electromagnetic waves as a treatment technique. RF diathermy is commonly utilized in therapy due to its thermal effects, primarily targeting pain and inflammation relief while also enhancing tissue extensibility [8]. However, the effectiveness of specific wave frequencies for musculoskeletal pain management remains unclear. The 4.4 MHz RF diathermy combines both capacitive and resistive electrical transfer mechanisms, enabling effective deep tissue penetration without causing muscle damage. The application of 4.4 MHz RF raises the muscle temperature without causing harm to cells, reduces muscle swelling, and exhibits anti-inflammatory effects within tissues [9]. Previous studies have demonstrated the efficacy of 4.4 MHz RF diathermy in managing pain, such as low back pain and shoulder pain [9,10]. While there have been studies on RF diathermy for KOA, the frequencies applied in each study have varied [11,12,13,14]. However, there are still not enough prospective studies performed to investigate the effectiveness of 4.4 MHz RF diathermy in treating KOA. The objective of this study is to investigate the clinical effectiveness of 4.4 MHz RF diathermy in terms of reducing pain, improving functional capacity, and enhancing the quality of life for patients. Additionally, this study aims to compare the efficacy of this treatment with the results of US treatment in individuals diagnosed with KOA.

## 2. Materials and Methods

### 2.1. Study Design and Participants

This prospective, double-blind, randomized controlled trial received approval from the institutional review board (IRB) of the authors’ affiliated institutions (Korea University Anam Hospital, Seoul, Republic of Korea; IRB protocol No. 2021AN0154; Ajou University Hospital, Suwon, Republic of Korea; IRB protocol No. AJOUIRB-DEV-2020-607). This study was also registered in the protocol registration system of the clinical research information service (https://cris.nih.go.kr/, KCT0006585, accessed on 9 August 2021). Before participating in this study, all individuals provided written informed consent.

This study included a total of 108 patients experiencing knee pain, who were recruited from pain clinics at two hospitals. Eligible patients included those aged 50–79 years who visited the pain clinic between October 2021 and October 2022 and were diagnosed with KOA.

We enrolled patients who gave written informed consent and met the following inclusion criteria: (1) conformance with the American College of Rheumatology classification criteria for knee osteoarthritis and (2) attainment of Kellgren and Lawrence (KL) grade 2 or 3. The following exclusion criteria were used for participant selection: (1) other causes of osteoarthritis (e.g., rheumatoid, gout, infectious); (2) history of knee joint replacement in the affected knee; (3) previous treatment (ultrasound therapy, extracorporeal shock wave therapy, or intra-articular injection) within the preceding six months; (4) history of surgery, trauma, or cancer in the affected knee; (5) significant cognitive or communication impairment that could hinder appropriate responses to the questionnaire; (6) contact dermatitis causing difficulty in using treatment modality or gel; (7) peripheral neuropathy; (8) pregnancy in women; and (9) additional conditions that might warrant ineligibility as determined through clinical assessment. All other treatments for pre-existing diseases (hypertension, hyperlipidemia, etc.) were permitted during this study except those that might affect the result of this study (changing painkiller doses, physical therapy, etc.).

### 2.2. Random Allocation and Blinding Method

Once a patient fulfilled all the eligibility criteria, they were assigned randomly to either the RF group or the US group using a block randomization technique. Enlisted patients were each given a random allocation code generated using the ‘Proc Plan’ command in SAS version 9.4 (SAS institute, Cary, NC, USA). A random allocation code was designated based on the order of participants registration, using the generated randomization table.

The allocation details of the patients were provided to the physical therapists at each hospital, following a double-blind method, without informing the assessors or patients. The researcher in charge of randomization remained unaware of the assignment information until the end of this study. If a patient stopped treatment, the number was not used again, and the patient was not eligible to reapply. For the blinding technique, all procedures were performed on a bed equipped with a barrier that completely blocked the view of the patient; the equipment was not exposed until the patient entered the barrier.

### 2.3. Intervention

**Figure 1:**
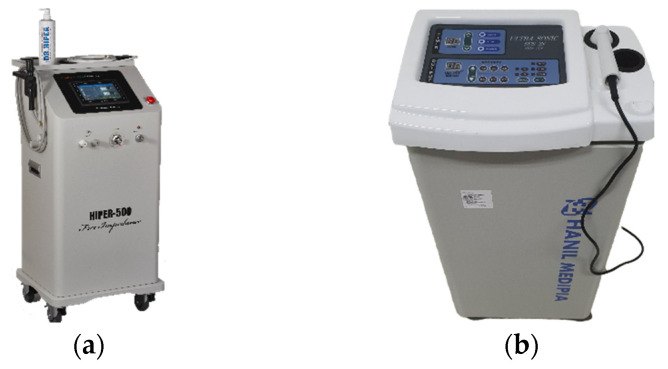
The apparatus employed for intervention in this study. (**a**) A transcutaneous radiofrequency diathermy device, HIPER-500. (**b**) A therapeutic ultrasound device, SUS-2N.

Both groups received treatment three times per week, with a total of 10 sessions over four weeks. In the course of each treatment session, the therapy was administered for a duration of 15 min. The RF group received treatment utilizing a HIPER-500 diathermy apparatus^®^ (JS-ON Corporation, Seoul, Republic of Korea), which applied a high level of 45 W/cm^2^ (±20%) of output energy (at 500 Ω) to the treatment area of the subjects using a polyamide-coated insulating electrode at a frequency of 4.4 MHz (Figure 1A). The electrode consisted of a probe with a 70 mm diameter positive electrode coupled to a 185 mm handpiece and a negative electrode with a size of 150 mm × 200 mm. Both electrodes were made of aluminum and coated with a polyamide insulator to ensure that the displacement current was evenly distributed over the electrode surface due to internal resistance through a combination of CET and RET methods. A conductive gel was applied between the positive electrode and the patient’s skin to improve conductivity. The US group received treatment utilizing ultrasound apparatus, Ultrasonic SUS-2N^®^ (SHIN JIN, Seoul, Republic of Korea), which applied a frequency of 3 MHz (Figure 1B). The maximum power output was 1.5 W/m^2^, and the intensity was expressed as a percentage. In both groups, the positive electrode probe was continuously moved and applied to the area around the knee to prevent excessive temperature rise or patient discomfort during the procedure. The electrodes connected to the RF or US apparatus and the external components of the RF and US devices were concealed with black opaque paper to ensure participants could not identify them. The duration of treatment application was consistent in both groups. In this study, RF or US treatment was individually administered to each group, and no concurrent treatments for KOA, such as physical therapy, were given simultaneously. The sole permitted concurrent treatment for KOA was the use of NSAIDs.

### 2.4. Outcome Measures

Patients underwent assessment during a total of four measurement sessions: baseline assessment, after the 5th treatment, after the 10th treatment, and four weeks following the conclusion of all treatment sessions. The primary measure for evaluating outcomes was the pain intensity. Pain intensity was gauged using a numeric rating scale (NRS) that spanned from 0 (no pain) to 10 (worst imaginable pain). Secondary outcomes were the Western Ontario and McMaster Universities Osteoarthritis Index (WOMAC) score, gait analysis, Lequesne index, and 36-Item Short Form Health Survey (SF-36). For WOMAC score and SF-36, we were able to find a Korean translation version widely used [15,16], but we could not find the Korean version of Lequesne index. Therefore, we translated the English version of the Lequesne index into Korean [17]. The WOMAC score evaluates 5 factors related to pain (with a score range of 0–20), 2 factors concerning stiffness (with a score range of 0–8), and 17 factors associated with functional limitations (with a score range of 0–68). Elevated scores indicate increased pain, stiffness, and functional limitations. Questions related to physical functions encompassed a range of daily activities, including using stairs, getting up from a seated or lying position, standing, bending, walking, getting in and out of a car, shopping, putting on or taking off socks, lying down in bed, entering or exiting a bath, sitting, and performing both heavy and light household tasks. For gait analysis, sensors (Legsys, BioSensics, Newton, MA, USA) were applied at the mid-height of both calves of the patients and a distance of 30 m was walked twice. It provided information on the stride length (m), percentage of height (%height), time (s), speed 1 (m/s), speed 2 (%height/s), and rhythm (strides/min). Lequesne index (LI) consists of 11 questions that evaluate (1) pain or discomfort, (2) maximum walking distance, and (3) activities of daily living. SF-36 encompasses a set of generic, coherent, and easily administered quality-of-life measures. SF-36 comprises eight scaled scores: vitality, physical functioning, bodily pain, general health perceptions, physical role functioning, emotional role functioning, social role functioning, mental health, and emotional well-being. Reduced scores indicate higher levels of disability. A higher score corresponds to a lower level of disability; specifically, a score of zero indicates maximum disability, while a score of 100 indicates no disability. While WOMAC score, Lequesne index, and gait analysis were assessed at baseline, after the 5th and 10th treatment and four weeks following the conclusion of all treatment sessions, SF-36 was evaluated only at the baseline and four weeks after the completion of all the treatments. Weight, vital signs, adverse effects, and drug intake (within one month of 1st visit) were recorded at every visit. During the initial assessment, sex, age, medication history within one year, and X-ray results were documented.

### 2.5. Statistical Analysis

In calculating the sample size, the reference study involving focal low-intensity pulsed ultrasound treatment for pain reduction in KOA patients was consulted. The difference in the average change in pain scores between groups (1.14) was taken as the assumed difference in the Numeric Rating Scale (NRS) change before and after, with a standard deviation set at 1.878. At a two-sided significance level of 5% and a power of 80%, the number of subjects calculated was 43 per group, and a total of 108 subjects (54 per group) were calculated considering the dropout rate of 20%.

Statistical analysis involved the utilization of the Student’s *t*-test for continuous variables, encompassing age, height, weight, pulse rate, blood pressure, Numeric Rating Scale (NRS), Lequesne index, WOMAC score, SF-36, stride length, stride velocity, stride time, and cadence. Categorical variables, including sex, were analyzed using the chi-square test or Fisher’s exact test. The data are presented as mean ± standard deviation for continuous variables, and as number (%) for categorical variables. Statistical significance was established at a *p*-value of less than 0.05. All statistical analyses were carried out using SAS (version 9.4; Cary, NC, USA) and R software (version 4.04; R Foundation for Statistical Computing, Vienna, Austria).

## 3. Results

**Figure 2:**
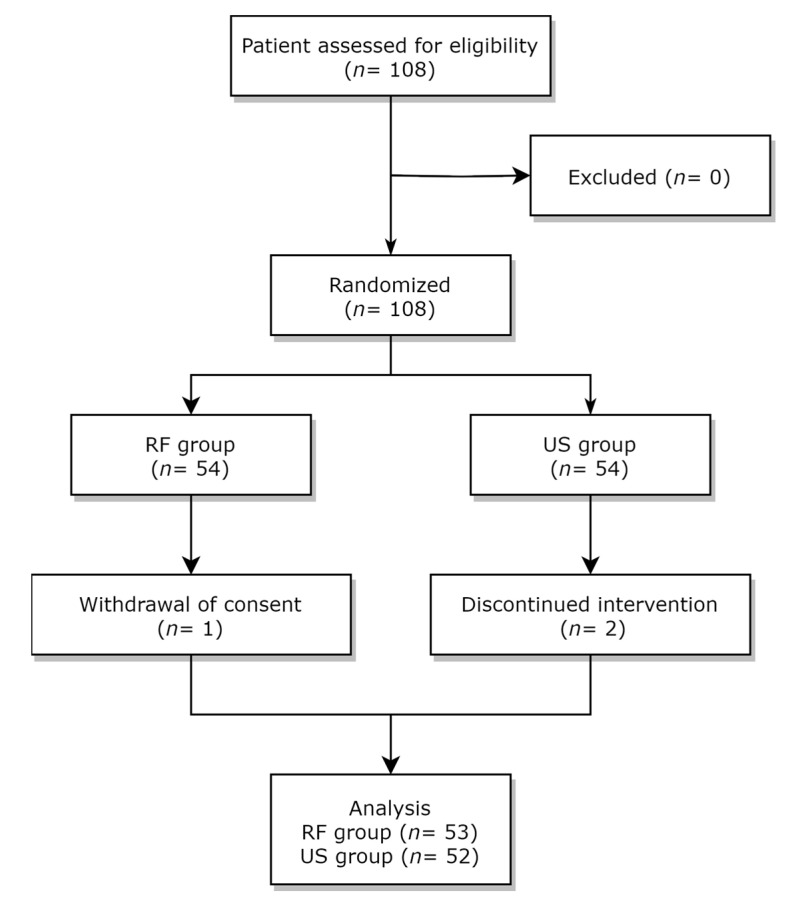
A flow diagram illustrating the study design. A total of 108 patients were randomly assigned to either the transcutaneous radiofrequency (RF) diathermy group or the therapeutic ultrasound (US) group.

A total of 108 patients were randomly allocated to either the RF group or the US group. After enrollment, there was a patient who changed their mind and withdrew consent before starting treatment in the RF group, and there were two patients who were isolated during treatment due to an infectious disease (COVID-19). All other patients completed four weeks of treatment (Figure 2).

Table 1 displays the demographic characteristics of the study’s enrolled participants. No significant disparities were found in the demographic data or baseline physical conditions between the two groups.

The changes in NRS scores in both groups are shown in Table 2. The initial NRS was 4.42 ± 1.18 and 4.56 ± 1.06 in the RF and US groups, respectively. Following the fifth treatment, the NRS in the RF group declined to 2.91 ± 1.39, and further to 2.23 ± 1.12 after the 10th treatment, reaching 1.98 ± 1.25 at four weeks post treatment. On the other hand, the NRS in the US group declined to 3.08 ± 1.12 after the fifth treatment, and further to 2.41 ± 1.31 after the 10th treatment, reaching 2.25 ± 1.48 at four weeks after treatment. Both the RF and US groups demonstrated a substantial decrease in NRS scores; however, there was no significant disparity in NRS scores between the two groups.

Changes in the WOMAC score and LI are presented in Table 3 and Table 4, respectively. As the treatment commenced, both the RF and US groups exhibited reductions in each component of the WOMAC scores (pain, stiffness, and activity), as well as in the total score. By the fourth-week post-treatment visit, both groups demonstrated significant decreases in the WOMAC scores across all parameters compared to baseline. However, for the WOMAC components of pain and stiffness, as well as the overall WOMAC score, there were higher scores at the 4-week post-treatment visit compared to the treatment endpoint, but these differences were not statistically significant in either group.

The initial LI was 7.25 ± 3.20 in the RF group and 6.38 ± 3.41 in the US group. After the fifth treatment, the LI in the RF group decreased to 6.16 ± 3.55 and further declined to 4.98 ± 3.38 after the 10th treatment. In the US group, the LI decreased to 5.43 ± 3.10 after the 5th treatment and further declined to 4.56 ± 3.21 after the 10th treatment. However, at the fourth-week follow-up post treatment, the LI slightly increased to 5.06 ± 3.31 in the RF group and 4.75 ± 3.77 in the US group. Nevertheless, both groups showed a significant reduction in LI compared to baseline values at the end of treatment and the fourth-week post-treatment follow-up. The increase in LI values after 4 weeks of treatment was not statistically significant when compared to the 10th treatment endpoint (*p* values for RF group and US group were 0.845 and 0.533, respectively). Furthermore, no significant differences were observed between the two groups at each time point.

The findings for the SF-36 are outlined in Table 5. No noteworthy differences were noted between the two groups concerning any of the SF-36 parameters at each evaluation interval. However, in the context of within-group comparisons to baseline, a notable improvement in the bodily pain parameter was observed exclusively within the RF group. On the other hand, a significant improvement in terms of general health and social functioning, compared to baseline, was evident solely within the US group. Both groups demonstrated improvements in bodily pain and mental health when compared to their respective baseline levels. Nevertheless, both groups showed substantial improvements in the total score when compared to their initial baselines.

The outcomes of gait analysis are detailed in Table 6. During gait analysis, both groups demonstrated an increasing trend in stride length as the number of treatments increased, compared to baseline. The RF group demonstrated a notable stride length increase at the conclusion of the treatment. Both groups exhibited a significant stride length augmentation at the four-week post-treatment follow-up when compared to baseline measurements. Stride time showed no significant differences compared to baseline in both groups. Both groups displayed an increasing trend in stride velocity compared to baseline, with the RF group showing significant increases at the treatment endpoint and the four-week post-treatment follow-up. The US group exhibited a significant increase in stride velocity at the four-week post-treatment follow-up. Solely the RF group demonstrated a significant increase in cadence at the four-week post-treatment assessment. Nonetheless, no significant distinctions were evident between the two groups for any of the gait analysis parameters at each assessment point.

## 4. Discussion

In this study, we conducted a comparison between the effectiveness of a newly developed deep heat therapy approach, 4.4 MHz RF diathermy, and therapeutic US treatment. Our aim was to alleviate pain and enhance functional recuperation in patients experiencing knee pain attributed to osteoarthritis. Both groups showed improvements in the NRS, WOMAC, LI, SF-36, and gait analysis parameters after 10 treatment sessions. These improvements were sustained for a period of four weeks following the conclusion of the therapy. A significant statistical difference between the two groups was not observed in most of the results.

Heat therapy regulates pain by reducing nociceptive activity, increasing blood flow, promoting muscle relaxation, and modulating inflammatory cytokines [18]. Generally, heat leads to increased chemical activity and metabolic rates in cells and tissues, resulting in vasodilation and increased blood flow. Increased blood flow facilitates tissue recovery by delivering nutrients, oxygen, leukocytes, and antibodies. Additionally, increased vascular permeability allows the removal of toxins and necrotic substances by incorporating phagocytes and macrophages into the lesions. Moreover, elevated temperatures suppress the activities of various enzymes involved in inflammatory responses, thereby promoting the inhibition of chronic inflammatory reactions, pain relief, and functional improvement. Furthermore, elevated temperatures decrease the sensory nerve conduction velocity of C-fibers, which transmit pain signals, and increase the pain threshold, thereby reducing the transmission of pain input and enhancing the analgesic effect [18,19].

In this study, we employed a 4.4 MHz RF diathermy, incorporating both capacitive electric transfer (CET) and resistive electric transfer (RET). CET mainly generates surface temperature elevations close to the electrodes and possesses limitations in efficiently transferring heat to deeper tissues. In contrast, RET is effective in alleviating pain within deeper tissues characterized by higher resistance, such as tendons, bones, and joint structures, including ligaments [20]. Efforts have been made to pursue the advantages of these two modalities into treatments for KOA. The limitation of low permeability of the capacitive method can be compromised by applying the energy using dielectric materials. This method can increase tissue permeability through the use of high-frequency electromagnetic signals. It has been reported to effectively alleviate pain, improve the range of motion, and enhance the quality of life among patients with KOA [11,21]. On the other hand, in clinical studies using a 448 kHz energy transfer through a combination of capacitive and monopolar modes, a reduction in postoperative pain and improvements in gait ability and range of motion have also been reported [13].

These therapeutic effects are thought to be related to the blood flow increase and cellular metabolic activity as well as a reduction in muscle tone and increase in tissue extensibility [13]. These effects may promote regenerative reactions and enable physical therapy to show long-term effects, but they may be temporary. Although not statistically significant in this study, the fact that the treatment effect showed a decreasing trend one month after the end of treatment may perhaps be related to this reason. Therefore, further studies on the long-term persistence of these therapeutic effects are encouraged.

Therapeutic effects of diathermy equipment may differ when using different frequencies. The HIPER-500 device, which generates energy between an active probe and an inert ceramic plate to ensure consistency, has the potential to deliver deep and uniform energy to the deep joint areas in patients with KOA. This device has demonstrated therapeutic effects in reducing muscle swelling and inducing an anti-inflammatory response in rats without damaging the skin and muscles [22]. In cadaver studies, applying high-frequency therapy to the knee showed the potential to deliver heat effects to the capsule and inside the joint, depending on the output level [20]. However, this study was necessary because applying it to cadavers could have different effects in humans depending on the presence of blood flow and other mechanisms of thermoregulation.

KOA-related pain is caused by several mechanisms. The neural structure of each tissue, including the bone, cartilage, bone marrow, and synovitis, is thought to be closely related to knee pain [23]. Efforts have been made to locate these nerves and relieve knee pain through injection or ablation. Nevertheless, given the intricate nerve control structure of the knee, this task proves to be highly challenging [24]. Sensory nerves that innervate the knee are thought to originate mainly from the femoral and sciatic nerves; however, at the knee level, they are divided into numerous intricate branches and spread over a wide range [25]. Therefore, targeting various knee tissues spread over a wider area may be more effective. In the present study, there was a gradual reduction in pain with the number of treatments. One plausible factor is believed to be the expansion in treatment coverage, which is attributed to its repetitive administration.

In this study, the treatments demonstrated effectiveness not only in reducing pain but also in enhancing functional improvement. The etiology of KOA can stem not only due to problems within the knee joint but also from tissues located outside the joint [26]. This restriction in functional capacity is impacted by factors beyond the context of a specific task, encompassing variables such as the time of day and environmental conditions. Therefore, evaluation tools such as WOMAC and LI were used to evaluate these functions. This approach is primarily rooted in the intricate regulation of the inflammatory response, governed by a diverse array of factors. The dynamics of this response encompass various cytokines, enzymes, hormones, and related elements that interact in a cyclic pattern, ultimately contributing to tissue repair. However, if these reactions do not lead to sufficient tissue repair and occur repeatedly, chronic pain occurs [27]. Heat therapy promotes these reactions, ultimately reducing inflammation and helping in the restoration of damaged tissue [18]. Hence, it can be inferred that the treatments used in this study may have the potential to facilitate functional enhancements when adequately administered.

In this study, no significant distinction was observed between the groups in relation to the NRS and WOMAC scores, both of which were evaluated based on the patients’ subjective experiences. On the other hand, gait analysis was performed based on more objective measurements in comparison to these tests. In this study, while the magnitude of differences was not substantial, significant increments in stride length, speed, and step count per minute were observed within the RF group. These results are meaningful because they objectively reflect the strength of the muscles used during walking, sense of balance, and limitations caused by pain. In other words, although there was no difference that the patients could feel subjectively, the effect of a more extensive and uniform energy delivery in the RF group may have improved gait function. While additional, more precise research is required, these findings could carry significant implications as a tool for objectively assessing the efficacy of subsequent osteoarthritis treatment investigations.

This study had several limitations. First, it focused only on patients with KOA who had attained KL grades 1 and 2, limiting the generalizability of the findings to patients with more severe KOA or higher KL grades. Second, patient satisfaction was not assessed despite its importance in evaluating treatment outcomes, as demonstrated in other studies. Future studies should incorporate patient satisfaction assessments to provide a more comprehensive understanding. In addition, this study did not verify whether a consistent intensity was used for both RF diathermy and ultrasound therapy. It is valuable to compare the results obtained at different intensity levels. Furthermore, this study was deficient in long-term follow-up data, which hindered the assessment of treatments’ enduring effects and sustainability. Conducting long-term follow-up studies is crucial for evaluating the lasting efficacy of treatment outcomes.

To address these limitations, future studies should involve larger sample sizes, incorporate patient satisfaction assessments, explore different intensity levels, and include long-term follow-up evaluations to enhance our understanding of treatment outcomes.

## 5. Conclusions

According to the result of our study, transcutaneous RF diathermy demonstrated an efficacy comparable to the therapeutic US in relieving pain and improving functional capacity in individuals with KOA. Further research is essential to enhance the efficacy of noninvasive KOA treatments.

## Figures and Tables

**Table 1:** Demographic characteristics of the patients.

		RF Group (*n* = 53)	US Group (*n* = 52)	*p*-Value
Age, yr		61.45 ± 5.05	60.85 ± 5.11	0.5420 ^(1)^
Sex				0.1625 ^(2)^
	Male	1 (1.89%)	4 (7.69%)	
	Female	52 (98.11%)	48 (92.31%)	
Height, cm		154.17 ± 5.42	154.96 ± 4.82	0.4324 ^(1)^
Weight, kg		56.82 ± 6.70	58.27 ± 7.06	0.2847 ^(1)^
Pulse rate, bpm		75.57 ± 7.61	77.50 ± 7.48	0.1921 ^(1)^
SBP, mmHg		127.51 ± 11.51	130.56 ± 11.85	0.1842 ^(1)^
DBP, mmHg		78.06 ± 8.59	78.56 ± 8.45	0.7638 ^(1)^

Data are presented as mean ± standard deviation or number (%); *p*-value assessed using Student’s *t*-test ^(1)^ and chi-square test ^(2)^. RF: radiofrequency. US: ultrasound. bpm: beats per minute. SBP: systolic blood pressure. DBP: diastolic blood pressure.

**Table 2:** Follow-up and alterations in the Numeric Rating Scale (NRS).

	RF Group (*n* = 53)	US Group (*n* = 52)	Within-Group Mean Difference		Between-Group Mean Difference	*p*-Value ^(1)^
			RF Group	US Group		
Visit 1	4.42 ± 1.18	4.56 ± 1.06			−0.16 [−0.60/0.27]	0.5165
Visit 2	2.91 ± 1.39	3.08 ± 1.12	−1.51 [−1.79/−1.23] **	−1.51 [−1.78/−1.24] **	−0.19 [−0.68/0.30]	0.4887
Visit 3	2.23 ± 1.12	2.41 ± 1.31	−2.19 [−2.48/−1.90] **	−2.12 [−2.47/−1.76] **	−0.24 [−0.72/0.25]	0.4401
Visit 4	1.98 ± 1.25	2.25 ± 1.48	−2.43 [−2.80/−2.07] **	−2.31 [−2.73/−1.90] **	−0.27 [−0.81/0.26]	0.3097

Data are presented as mean ± standard deviation, mean difference [95% confidence interval]; ^(1)^ *p*-value evaluated using Student’s *t*-test between groups. Visit 1: before the start of the treatment (baseline evaluation); visit 2: after the 5th treatment; visit 3: after the 10th treatment; and visit 4: four weeks post treatment. RF: radiofrequency. US: ultrasound. ** Indicates statistically significant within-group differences compared to visit 1 (*p* < 0.001).

**Table 3:** Follow-up and alterations in the WOMAC score.

		RF Group (*n* = 53)	US Group (*n* = 52)	Within-Group Mean Difference		Between-Group Mean Difference	*p*-Value ^(1)^
				RF Group	US Group		
Pain	Visit 1	5.53 ± 2.97	4.87 ± 3.41			0.59 [−0.66/1.83]	0.2902
	Visit 2	4.34 ± 3.06	3.50 ± 2.80	−1.19 [−2.11/−0.26] *	−1.37 [−2.4/−0.35] *	0.76 [−0.38/1.91]	0.1462
	Visit 3	3.26 ± 2.70	2.73 ± 2.75	−2.26 [−3.08/−1.45] **	−2.13 [−2.97/−1.30] **	0.46 [−0.61/1.52]	0.3157
	Visit 4	3.87 ± 3.15	3.29 ± 3.12	−1.66 [−2.76/−0.56] *	−1.59 [−2.56/−0.62] *	0.57 [−0.65/1.79]	0.3530
Stiffness	Visit 1	2.09 ± 1.60	1.54 ± 1.51			0.56 [−0.05/1.16]	0.0701
	Visit 2	1.60 ± 1.45	1.10 ± 1.45	−0.49 [−0.99/0.01]	−0.47 [−1.02/0.08]	0.55 [−0.01/1.11]	0.0749
	Visit 3	1.23 ± 1.32	0.88 ± 1.13	−0.87 [−1.35/−0.38] **	−0.60 [−1.09/−0.10] *	0.28 [−0.21/0.77]	0.1573
	Visit 4	1.30 ± 1.55	1.06 ± 1.41	−0.79 [−1.26/−0.32] **	−0.49 [−0.94/−0.04] *	0.24 [−0.33/0.82]	0.4050
Activity	Visit 1	17.47 ± 11.51	15.69 ± 11.82			1.38 [−3.17/5.92]	0.4362
	Visit 2	13.06 ± 9.85	10.13 ± 8.16	−4.42 [−7.91/−0.92] *	−6.02 [−8.82/−3.22] **	2.79 [−0.78/6.35]	0.1012
	Visit 3	10.53 ± 9.49	9.67 ± 9.01	−6.94 [−10.10/−3.79] **	−5.90 [−8.82/−2.99] **	0.34 [−3.38/4.05]	0.6362
	Visit 4	10.11 ± 10.08	9.61 ± 9.67	−7.36 [−10.70/−4.01] **	−6.20 [−9.36/−3.03] **	0.51 [−3.34/4.35]	0.7949
Total	Visit 1	25.09 ± 14.66	22.10 ± 15.22			2.52 [−3.3/8.33]	0.3062
	Visit 2	19.00 ± 13.53	14.73 ± 10.94	−6.09 [−10.45/−1.74] *	−7.86 [−11.67/−4.06] **	4.10 [−0.73/8.93]	0.0787
	Visit 3	15.02 ± 12.54	13.27 ± 12.23	−10.08 [−14.03/−6.12] **	−8.63 [−12.33/−4.94] **	1.08 [−3.87/6.03]	0.4746
	Visit 4	15.28 ± 13.64	13.96 ± 13.31	−9.81 [−14.23/−5.39] **	−8.27 [−12.36/−4.18] **	1.32 [−3.92/6.57]	0.6182

Data are presented as mean ± standard deviation, mean difference [95% confidence interval]; *p*-value evaluated using ^(1)^ Student’s *t*-test between groups. Visit 1: before the start of the treatment (baseline evaluation); visit 2: after the 5th treatment; visit 3: after the 10th treatment; and visit 4: four weeks post treatment. * Indicates statistically significant within-group differences compared to visit 1 (*p* < 0.05); ** Indicates statistically significant within-group differences compared to visit 1 (*p* < 0.001).

**Table 4:** Follow-up and alterations in the Lequesne index.

	RF Group (*n* = 53)	US Group (*n* = 52)	Within-Group Mean Difference		Between-Group Mean Difference	*p*-Value ^(1)^
			RF Group	US Group		
Visit 1	7.25 (3.20)	6.38 (3.41)			0.87 [−0.41/2.15]	0.1847
Visit 2	6.16 (3.55)	5.43 (3.10)	−1.08 [−2.01/−0.16] *	−0.95 [−1.75/−0.16] *	0.66 [−0.64/1.96]	0.2661
Visit 3	4.98 (3.38)	4.56 (3.21)	−2.26 [−3.21/−1.32] **	−1.75 [−2.43/−1.07] **	0.36 [−0.92/1.63]	0.5152
Visit 4	5.06 (3.31)	4.75 (3.77)	−2.19 [−3.2/−1.17] **	−1.64 [−2.48/−0.8] **	0.30 [−1.08/1.68]	0.6652

Data are presented as mean ± standard deviation, mean difference [95% confidence interval]; *p*-value evaluated using ^(1)^ Student’s *t*-test between groups. Visit 1: before the start of the treatment (baseline evaluation); visit 2: after the 5th treatment; visit 3: after the 10th treatment; and visit 4: four weeks post treatment. * Indicates statistically significant within-group differences compared to visit 1 (*p* < 0.05); ** Indicates statistically significant within-group differences compared to visit 1 (*p* < 0.001).

**Table 5:** Follow-up and alterations in the SF-36 scores.

		RF Group (*n* = 53)	US Group (*n* = 52)	Within-Group Mean Difference		Between-Group Mean Difference	*p*-Value ^(1)^
				RF Group	US Group		
Physical functioning	Visit 1	62.08 ± 21.27	66.15 (21.39)			−4.18 [−12.42/4.07]	0.3295
	Visit 4	69.53 ± 21.29	72.16 (18.36)	7.45 [0.51/14.39] *	5.69 [−0.23/11.6]	−2.63 [−10.37/5.12]	0.5024
Role limitation-physical	Visit 1	61.79 ± 29.66	64.42 ± 31.45			−2.15 [−13.99/9.69]	0.6601
	Visit 4	67.45 ± 32.74	70.59 ± 32.67	5.66 [−3.57/14.89]	6.37 [−1.31/14.05]	−3.14 [−15.86/9.59]	0.6261
Bodily pain	Visit 1	64.43 ± 17.35	69.76 ± 13.69			−5.52 [−11.58/0.54]	0.0841
	Visit 4	74.95 ± 16.67	76.52 ± 17.59	10.52 [4.49/16.54] *	6.72 [1.77/11.66] *	−1.57 [−8.23/5.1]	0.6419
General health	Visit 1	53.87 ± 15.37	53.46 ± 16.79			0.5 [−5.74/6.74]	0.8973
	Visit 4	56.70 ± 15.87	57.65 ± 18.61	2.83 [−1.18/6.84]	4.02 [0.72/7.32] *	−0.95 [−7.67/5.77]	0.7799
Vitality	Visit 1	54.91 ± 17.31	57.50 ± 14.87			−2.4 [−8.66/3.85]	0.4123
	Visit 4	57.36 ± 14.86	60.20 ± 18.22	2.45 [−1.98/6.88]	2.75 [−1.78/7.27]	−2.84 [−9.29/3.62]	0.3853
Social functioning	Visit 1	67.45 ± 18.24	68.51 ± 17.06			−0.82 [−7.65/6.02]	0.7598
	Visit 4	68.87 ± 19.71	72.06 ± 17.96	1.42 [−3.98/6.81]	3.68 [−1.78/9.14] *	−3.19 [−10.53/4.15]	0.3906
Role limitation-emotion	Visit 1	69.18 ± 38.59	78.22 ± 32.93			−9.03 [−22.93/4.87]	0.2004
	Visit 4	79.25 ± 35.34	86.28 ± 27.63	10.06 [−0.98/21.11]	8.49 [−2.89/19.87]	−7.03 [−19.4/5.34]	0.2623
Mental health	Visit 1	63.47 ± 16.19	66.92 ± 14.33			−3.53 [−9.45/2.4]	0.2504
	Visit 4	68.60 ± 15.20	71.14 ± 15.95	5.13 [1.16/9.1] *	4.24 [0.88/7.59] *	−2.53 [−8.59/3.52]	0.4087
Total	Visit 1	497.18 ± 125.01	524.95 ± 106.71			−27.12 [−72.14/17.91]	0.2242
	Visit 4	542.71 ± 114.34	566.58 ± 114.93	45.53 [13.89/77.16]	41.94 [14.04/69.84] *	−23.87 [−68.47/20.73]	0.2909

Data are presented as mean ± standard deviation, mean difference [95% confidence interval]; *p*-value evaluated using ^(1)^ Student’s *t*-test between groups. Visit 1: before the start of the treatment (baseline evaluation); visit 2: after the 5th treatment; visit 3: after the 10th treatment; and visit 4: four weeks post treatment. * Indicates statistically significant within-group differences compared to visit 1 (*p* < 0.05).

**Table 6:** Follow-up and alterations in gait analysis.

		RF Group (*n* = 53)	US Group (*n* = 52)	Within-Group Mean Difference		Between-Group Mean Difference	*p*-Value ^(1)^
				RF Group	US Group		
Stride length (cm)	Visit 1	1.16 ± 0.19	1.18 ± 0.16			−0.02 [−0.08/0.05]	0.7047
	Visit 2	1.19 ± 0.17	1.18 ± 0.17	0.02 [−0.02/0.07]	0.02 [−0.01/0.05]	−0.02 [−0.08/0.04]	0.9740
	Visit 3	1.22 ± 0.11	1.21 ± 0.12	0.06 [0.01/0.11] *	0.03 [−0.01/0.07]	0.02 [−0.03/0.06]	0.4361
	Visit 4	1.23 ± 0.10	1.24 ± 0.13	0.06 [0.02/0.11] *	0.06 [0.02/0.1] *	−0.02 [−0.06/0.03]	0.4321
%Stride length (%height)	Visit 1	75.45 ± 11.96	76.19 ± 9.80			−0.73 [−4.97/3.5]	0.8014
	Visit 2	76.78 ± 10.49	77.81 ± 8.84	1.33 [−1.56/4.21]	1.23 [−0.92/3.38]	−1.03 [−4.79/2.73]	0.9057
	Visit 3	79.41 ± 7.63	78.07 ± 7.85	3.99 [0.84/7.14] *	1.88 [−0.56/4.32]	1.34 [−1.67/4.35]	0.3901
	Visit 4	79.49 ± 6.67	79.59 ± 7.81	4.04 [1.05/7.03] *	3.29 [0.76/5.82] *	−0.10 [−2.92/2.72]	0.9442
Stride time (sec)	Visit 1	1.03 ± 0.07	1.03 ± 0.08			0.01 [−0.02/0.04]	0.5335
	Visit 2	1.03 ± 0.07	1.04 ± 0.08	−0.01 [−0.02/0.01]	0.01 [−0.01/0.03]	−0.01 [−0.04/0.02]	0.4924
	Visit 3	1.03 ± 0.06	1.03 ± 0.07	0.00 [−0.02/0.02]	0.00 [−0.02/0.02]	0.01 [−0.02/0.03]	0.6223
	Visit 4	1.02 ± 0.07	1.02 ± 0.07	−0.02 [−0.03/0]	0.00 [−0.02/0.02]	0.00 [−0.03/0.02]	0.7332
Stride velocity (cm/s)	Visit 1	1.14 ± 0.20	1.16 ± 0.18			−0.03 [−0.1/0.05]	0.6472
	Visit 2	1.18 ± 0.19	1.17 ± 0.21	0.03 [−0.01/0.08]	0.02 [−0.02/0.06]	−0.01 [−0.08/0.06]	0.8277
	Visit 3	1.21 ± 0.14	1.21 ± 0.14	0.07 [0.02/0.12] *	0.04 [−0.01/0.08]	0.01 [−0.05/0.06]	0.8687
	Visit 4	1.23 ± 0.13	1.23 ± 0.15	0.08 [0.03/0.13] *	0.07 [0.02/0.11] *	−0.01 [−0.06/0.05]	0.8151
%Stride velocity (%)	Visit 1	74.19 ± 13.38	75.79 ± 11.73			−1.60 [−6.47/3.28]	0.6774
	Visit 2	76.27 ± 12.16	76.88 ± 11.38	2.08 [−0.95/5.11]	1.07 [−1.28/3.43]	−0.61 [−5.17/3.95]	0.7810
	Visit 3	78.66 ± 9.56	77.89 ± 9.32	4.47 [1.19/7.75] *	2.1 [−0.92/5.12]	0.77 [−2.89/4.42]	0.7430
	Visit 4	79.55 ± 8.86	79.6 ± 9.45	5.36 [2.07/8.64] *	4.06 [1.15/6.96] *	−0.05 [−3.61/3.51]	0.9783
Cadence (steps/min)	Visit 1	115.98 ± 7.11	115.99 ± 7.52			−0.12 [−2.93/2.7]	0.9991
	Visit 2	116.94 ± 7.65	115.90 ± 7.78	0.95 [−0.39/2.29]	−0.14 [−1.85/1.58]	0.95 [−2.03/3.93]	0.4913
	Visit 3	116.69 ± 7.20	117.63 ± 7.42	0.7 [−0.74/2.14]	1.31 [−0.21/2.84]	−0.73 [−3.57/2.12]	0.5141
	Visit 4	118.34 ± 7.80	117.67 ± 7.37	2.35 [0.53/4.17] *	1.53 [−0.13/3.19]	0.67 [−2.29/3.62]	0.6553

Data are presented as mean ± standard deviation, mean difference [95% confidence interval]; *p*-value evaluated using ^(1)^ Student’s *t*-test between groups. Visit 1: before the start of the treatment (baseline evaluation); visit 2: after the 5th treatment; visit 3: after the 10th treatment; and visit 4: four weeks post treatment. * Indicates statistically significant within-group differences compared to visit 1 (*p* < 0.05).

## Data Availability

The datasets utilized and/or analyzed during the present study are accessible from the corresponding author upon reasonable request.
